# BRG1 promotes survival of UV-irradiated melanoma cells by cooperating with MITF to activate the melanoma inhibitor of apoptosis gene

**DOI:** 10.1111/pcmr.12088

**Published:** 2013-03-08

**Authors:** Srinivas V Saladi, Philip G Wong, Archit R Trivedi, Himangi G Marathe, Bridget Keenen, Shweta Aras, Zi-Qi Liew, Vijayasaradhi Setaluri, Ivana L de la Serna

**Affiliations:** 1 Department of Biochemistry and Cancer Biology, University of Toledo College of MedicineToledo, OH, USA; 2 Department of Dermatology, University of WisconsinMadison, WI, USA

**Keywords:** melanoma, MITF, SWI/SNF enzymes, chromatin remodeling, ultraviolet radiation, apoptosis, ML-IAP

## Abstract

Microphthalmia-associated transcription factor (MITF) is a survival factor in melanocytes and melanoma cells. MITF regulates expression of antiapoptotic genes and promotes lineage-specific survival in response to ultraviolet (UV) radiation and to chemotherapeutics. SWI/SNF chromatin-remodeling enzymes interact with MITF to regulate MITF target gene expression. We determined that the catalytic subunit, BRG1, of the SWI/SNF complex protects melanoma cells against UV-induced death. BRG1 prevents apoptosis in UV-irradiated melanoma cells by activating expression of the melanoma inhibitor of apoptosis (ML-IAP). Down-regulation of ML-IAP compromises BRG1-mediated survival of melanoma cells in response to UV radiation. BRG1 regulates ML-IAP expression by cooperating with MITF to promote transcriptionally permissive chromatin structure on the ML-IAP promoter. The alternative catalytic subunit, BRM, and the BRG1-associated factor, BAF180, were found to be dispensable for elevated expression of ML-IAP in melanoma cells. Thus, we illuminate a lineage-specific mechanism by which a specific SWI/SNF subunit, BRG1, modulates the cellular response to DNA damage by regulating an antiapoptotic gene and implicate this subunit of the SWI/SNF complex in mediating the prosurvival function of MITF.

## Introduction

Melanocytes synthesize and distribute melanin to surrounding cells on the skin, thus protecting against the damaging effects of ultraviolet (UV) radiation. Exposure to UV radiation causes DNA damage and is an environmental risk factor for developing melanoma (Jhappan et al., 2003). Malignant melanoma is refractory to chemotherapeutics and has a high mortality rate. The aggressive nature of melanoma is linked to expression of lineage-specific factors that are not present in other cell types (Gupta et al., 2005) and to the evolution of prosurvival mechanisms that render melanocytes resistant to death from UV radiation (Jhappan et al., 2003).

**Significance**

SWI/SNF enzymes interact with the microphthalmia-associated transcription factor (MITF), a lineage addiction oncogene, to promote MITF target gene expression in melanoma cells. In this study, we determined that the SWI/SNF component, BRG1, promotes melanoma survival in response to UV radiation, by activating expression of the melanoma inhibitor of apoptosis, ML-IAP gene. Our data show that BRG1 and MITF cooperate to establish permissive chromatin structure on the ML-IAP promoter and alter the association of other epigenetic regulators. Thus, we have elucidated a mechanism by which a component of the SWI/SNF complex promotes the prosurvival function of MITF. We further demonstrate that the BRG1-associated factor, BAF180, is not required for the activation of ML-IAP, suggesting that a specific configuration of the SWI/SNF complex mediates distinct activities. These results provide insight into how SWI/SNF function is deregulated in melanoma.

The microphthalmia-associated transcription factor (MITF) specifies the melanocyte lineage and promotes melanocyte survival. MITF is a lineage addiction oncogene that is amplified in about 20% of melanomas and contributes to melanoma chemoresistance (Garraway et al., 2005). MITF activates expression of the prosurvival genes, ML-IAP (BIRC7, livin) and BCL2 (Dynek et al., 2008; McGill et al., 2002). High levels of ML-IAP and BCL2 correlate with resistance to apoptosis following UV irradiation and treatment with other DNA-damaging agents (Bowen et al., 2003; Hornyak et al., 2009).

SWI/SNF enzymes are multisubunit complexes that remodel chromatin structure in an ATP-dependent manner and promote MITF target gene expression (de la Serna et al., 2006b; Keenen et al., 2010). Heterogeneous complexes are formed by the inclusion of one catalytic subunit, which is either BRG1 or BRM, and 8-12 associated factors (BAFs) (Keenen et al., 2010). Mammalian SWI/SNF complexes have been categorized as BAF and PBAF complexes (Yan et al., 2005). The BAF complex contains either BRG1 or BRM as the catalytic subunit and includes ARID1a or ARID1b among the associated factors. The PBAF complex contains only BRG1 as the catalytic subunit and includes at least two unique subunits: ARID2 and BAF180 (Yan et al., 2005). Components of the PBAF complex are mutated or down-regulated in several cancers, including melanoma, and may have a tumor-suppressive function (Decristofaro et al., 2001; Hodis et al., 2012; Varela et al., 2011; Xia et al., 2008).

In this study, we determined that BRG1 promotes survival of melanoma cells that have been exposed to UV radiation. We found that BRG1 protects melanoma cells from UV-induced death by stably activating expression of the melanoma inhibitor of apoptosis (ML-IAP, livin, BIRC7) gene. Our data show that activation of ML-IAP by BRG1 is highly dependent on MITF but not on the BRG1-associated factor, BAF180. BRG1 and MITF cooperate to establish permissive chromatin structure on the ML-IAP promoter and ensure high levels of ML-IAP expression. Interestingly, activation of ML-IAP is associated with increased histone acetylation and decreased levels of a repressive histone methylation mark. Consistent with this alteration in histone marks, there is increased recruitment of the histone acetyltransferase, CBP, and decreased recruitment of the EZH2 component of the polycomb complex. Thus, we have elucidated a mechanism by which a component of the SWI/SNF complex promotes the prosurvival function of MITF by remodeling chromatin structure on the promoter of an inhibitor of apoptosis gene.

## Results

### BRG1 protects melanoma cells from apoptosis after UV irradiation

**Figure 1:**
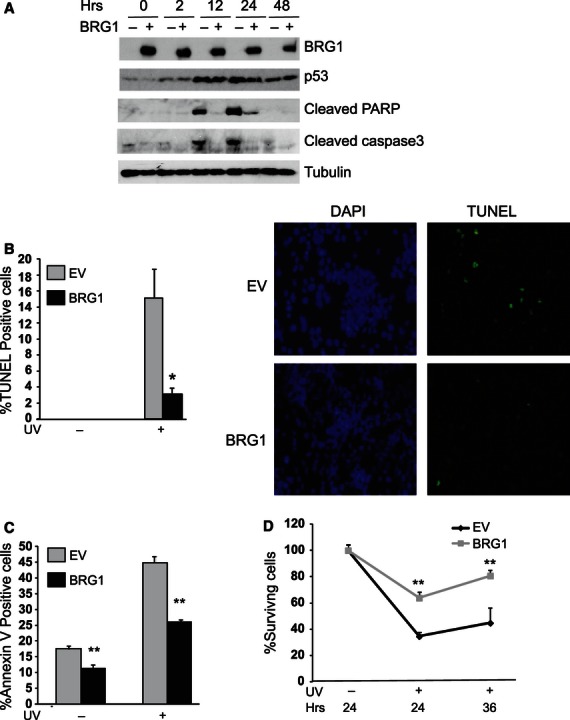
BRG1 protects melanoma cells from UV-induced apoptosis. (A) SK-MEL-5 cells stably expressing empty vector (EV) or BRG1 were sham-irradiated (Time 0) or irradiated with a UVB lamp. Cell extracts were prepared 0, 2, 12, 24, and 48 h after irradiation and subjected to Western blotting with antibodies to BRG1, p53, cleaved PARP, and cleaved caspase 3. Tubulin is a loading control. The data are representative of at least three independent experiments. (B) SK-MEL 5 cells were treated as in A and subjected to the TUNEL assay 36 h after irradiation. TUNEL-positive cells were counted in at least three fields and are expressed as percent of DAPI-stained cells (left). TUNEL-positive cells were not detected in sham-irradiated samples. Representative images of UV-irradiated cells are shown (right). The difference between TUNEL-positive (EV) and BRG1 cells was significant (P < 0.05). (C) SK-MEL-5 cells treated as in A were subjected to the annexin V assay 48 h after irradiation. Annexin V–positive cells were detected with a Guava Personal Cell Analysis System. There was a significant increase in annexin V–positive cells (P < 0.01) in UV-irradiated samples compared with sham-irradiated samples. BRG1 also significantly reduced the percent annexin V–positive cells in both sham- and UV-irradiated samples (P < 0.01). (D) Equal numbers of SK-MEL-5 cells expressing EV or BRG1 were plated and treated as in A and then counted at the indicated time points. Percent (%) surviving cells is relative to sham-irradiated cells taken at 24 h. The percent of BRG1-expressing cells that survived UV irradiation was significantly greater than the percent of control (EV) cells that survived (P < 0.01). The data (B–D) are the average of one experiment and representative of two independent experiments performed in triplicate. Standard deviations are shown.

SK-MEL-5 cells were previously determined to be deficient in BRG1 (Keenen et al., 2010). We constructed SK-MEL-5 cells that stably express BRG1 and found that BRG1 promotes expression of a subset of MITF target genes and increased resistance to the DNA-damaging agent, cisplatin (Keenen et al., 2010). To determine whether BRG1 also protects against UV-induced DNA damage, we irradiated control SK-MEL-5 cells (EV) and SK-MEL-5 cells expressing BRG1. The transcriptional regulator, p53, accumulated to similar levels in control and BRG1-expressing cells, beginning at 2 h after UV irradiation and reached a maximum level by 12 h after irradiation (Figure 1A). In control cells that lack BRG1, cleaved caspase 3 and cleaved PARP were detectable between 12 and 24 h following UV irradiation, decreasing by 48 h, as surviving cells presumably recovered from UV irradiation (Figure 1A). The levels of cleaved caspase 3 and cleaved PARP were strikingly lower in UV-irradiated BRG1-expressing cells than control cells at these time points. These data suggest that UV irradiation elicited a DNA damage response in control and BRG1-expressing melanoma cells and that BRG1 protected these cells from caspase dependent apoptosis.

We also performed a TUNEL assay on sham and UV-irradiated SK-MEL-5 cells that lack or express BRG1. We detected TUNEL-positive cells in UV-irradiated samples but not in sham-irradiated controls (data not shown). UV-irradiated BRG1-expressing cells had a reduced number of TUNEL-positive cells compared with UV-irradiated control cells lacking BRG1 (Figure 1B).

Because the TUNEL assay stains only adherent cells, we also performed an annexin V assay to quantify both adherent and floating cells undergoing apoptosis. BRG1 had a significant effect on the percent annexin V–positive cells even when cells were sham-irradiated (Figure 1C). UV irradiation significantly increased the number of annexin V–positive cells in both control (EV) and BRG1-expressing samples; however, the increase in annexin V–positive cells was significantly attenuated by BRG1 (Figure 1C). Furthermore, cell counts confirmed that the number of BRG1-expressing cells surviving UV irradiation was significantly greater than the number of surviving cells lacking BRG1 (Figure 1D). In combination, these data indicate that BRG1 protects melanoma cells to some extent from apoptosis during steady-state conditions and to a greater extent from apoptosis after UV irradiation.

### BRG1 promotes expression of the melanoma inhibitor of apoptosis (ML-IAP) gene

**Figure 2:**
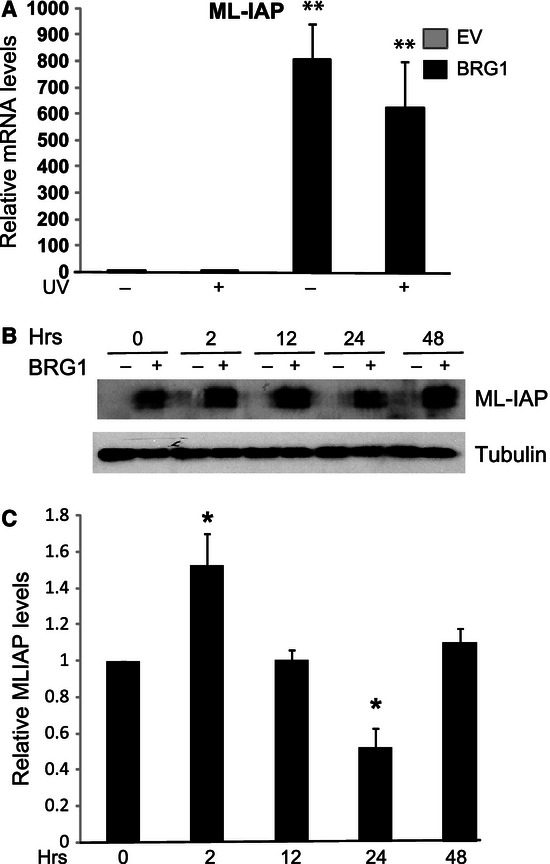
BRG1 activates ML-IAP expression in SK-MEL-5 melanoma cells. (A) Total RNA from sham- and UV-irradiated cells were harvested 24 h later, reverse-transcribed, and subjected to qPCR with primers to ML-IAP and GAPDH. There was a significant increase in ML-IAP mRNA in sham-irradiated SK-MEL-5 cells expressing BRG1 compared with sham-irradiated control SK-MEL-5 cells (EV) (P < 0.01), and there was a significant increase in ML-IAP in UV-irradiated SK-MEL-5 cells expressing BRG1 compared with UV-irradiated control SK-MEL-5 cells (EV) (P < 0.01). The data shown are the average of 3 or more independent experiments that were subjected to qPCR twice. Standard error bars are shown. (B) Cell extracts from SK-MEL-5 cells processed as in Figure 1A were subjected to Western blotting with an antibody to ML-IAP. Tubulin is a loading control. (C) The cell extracts from B were subjected to three Western blots and quantified by densitometry using Image J software. ML-IAP levels in BRG1-expressing cells are relative to the levels of tubulin. There was a significant increase in the level of ML-IAP at 2 h, and there was a significant decrease in the level of ML-IAP at 24 h relative to the level at 0 h (P < 0.05). Standard deviations are shown.

To understand the mechanisms by which BRG1 promotes survival in response to UV radiation, we investigated the requirement for BRG1 in the regulation of the melanoma inhibitor of apoptosis, ML-IAP. Restoration of BRG1 in SK-MEL-5 cells resulted in a dramatic increase in ML-IAP mRNA levels that was not further activated by exposure to UV radiation at the time points investigated (Figure 2A). At the protein level, the expression of two isoforms of ML-IAP, ML-IAPα, and ML-IAPβ was detected in BRG1-expressing cells at all time points but not in cells that lacked BRG1 (Figure 2B). We detected a transient increase in ML-IAP protein expression 2 h following exposure to UV radiation in BRG1-expressing cells (Figure 2B, C). Thus, BRG1 constitutively activates the expression of a potent inhibitor of apoptosis in SK-MEL-5 melanoma cells and may also be involved in transient activation of ML-IAP expression by UV radiation.

### BRG1-mediated protection of melanoma cells from UV-induced apoptosis is dependent on ML-IAP

**Figure 3:**
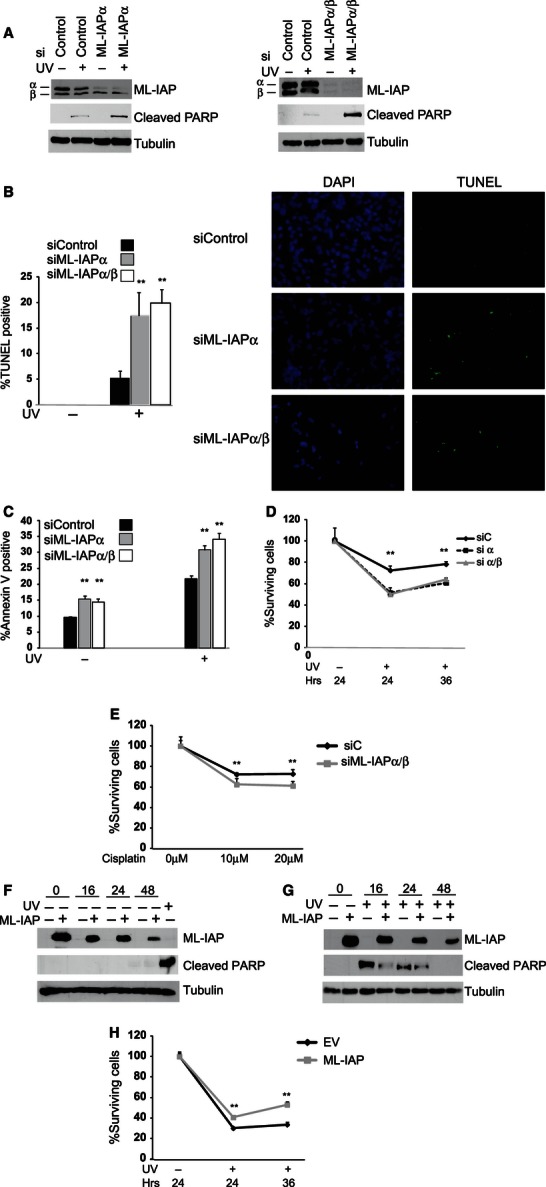
BRG1-mediated protection from UV-induced apoptosis is dependent on ML-IAP. (A) Expression of ML-IAPα (left) or both isoforms of ML-IAPα/β (right) was down-regulated with specific siRNAs or with a control-scrambled siRNA. SK-MEL-5 cells expressing BRG1 were sham- or UV-irradiated 72 h after transfection of siRNAs and harvested 36 h after UV irradiation. Total cell extracts were subjected to Western blotting with antibodies to ML-IAP and cleaved PARP. Tubulin is a loading control. The data are representative of two independent experiments. (B) Cells treated as in A were harvested 36 h after UV irradiation and subjected to the TUNEL assay. TUNEL-positive cells were counted in at least three fields and are expressed as percent of DAPI-stained cells. TUNEL-positive cells were not detected in sham-irradiated samples. Down-regulation of ML-IAP by either siML-IAPα or siML-IAPα/β resulted in a significant increase in TUNEL-positive cells compared with transfection with control siRNA (P < 0.05). The data are the average of one experiment and representative of two independent experiments performed in triplicate. Standard deviations are shown. Representative images of UV-irradiated cells are shown (right). (C) Cells treated as in A were harvested 48 h after UV irradiation and subjected to the annexin V assay. Down-regulation of ML-IAP by either siML-IAPα or siML-IAPα/β resulted in a significant increase in the percent annexin V–positive cells compared with transfection with control siRNA (P < 0.05) in both sham- and UV-irradiated samples. The data are the average of one experiment and representative of two independent experiments performed in triplicate. Standard deviations are shown. (D) SK-MEL-5 cells expressing BRG1 were transfected with a control siRNA, siML-IAPα, or siML-IAPα/β, treated as in A and counted at the indicated time points. Percent (%) surviving cells is relative to the number of corresponding sham-irradiated cells taken at 24 h. Percent survival was significantly lower in ML-IAP knockdown cells compared with control cells (P < 0.01). The data are the average of two independent experiments performed in triplicate. Standard deviations are shown. (E) Equal numbers of SK-MEL-5 cells expressing BRG1 were transfected with a control siRNA or siML-IAPα/β and treated with 10 or 20 μμ cisplatin. Cells were counted 48 h after treatment and normalized to untreated cells at the same time point. (F and G) SK-MEL-5 cells were transiently transfected with empty vector (EV) or cDNA-encoding ML-IAP and sham- or UV-irradiated 48 h after transfection. Protein extract was prepared from sham-irradiated cells (F) and UV-irradiated cells (G) at the indicated time point after irradiation and subjected to Western blotting with antibodies to ML-IAP and cleaved PARP. Tubulin is shown as a loading control. The last lane in F was prepared from UV-irradiated cells that express EV and serves as a positive control for cleaved PARP. H. Equal numbers of SK-MEL-5 cells transfected with ML-IAP cDNA or an empty vector (EV) were plated and treated as in G. Cell counts were taken at the indicated time points and normalized to the corresponding sham-irradiated controls. Transfection with ML-IAP significantly increased percent survival compared with transfection with an empty vector at both time points (P < 0.01). The data are the average of two independent experiments performed in triplicate. Standard deviations are shown.

The melanoma inhibitor of apoptosis (ML-IAP) is an MITF target gene that promotes melanoma survival. ML-IAP rescues melanoma viability in MITF-disrupted melanoma cells and can promote survival of malignant cells by intrinsic stress as well as in response to chemotherapeutics and other elicitors of DNA damage (Crnkovic-Mertens et al., 2003; Dynek et al., 2008; Liu et al., 2007). To determine whether the BRG1-mediated protection of SK-MEL-5 cells from death following UV irradiation is dependent on activation of ML-IAP, we down-regulated ML-IAP expression using an siRNA that targets ML-IAPα (Figure 3A, left panel) as well as an siRNA that targets both ML-IAP isoforms (Figure 3A, right panel). Knockdown of ML-IAPα or of both ML-IAPα and ML-IAPβ in BRG1-expressing SK-MEL-5 cells resulted in increased accumulation of cleaved PARP upon UV irradiation. Furthermore, knockdown of ML-IAP α and knockdown of both ML-IAP isoforms resulted in a significant increase in the percent TUNEL-positive cells detected after UV irradiation (Figure 3B). Annexin V staining indicated that knockdown of either α or both isoforms of ML-IAP significantly increased apoptosis of sham-irradiated samples and to a greater extent of UV-irradiated samples (Figure 3C). The number of BRG1-expressing melanoma cells that survived following exposure to UV radiation was also significantly decreased by knockdown of either ML-IAPα or both isoforms of ML-IAP (Figure 3D). Furthermore, knockdown of ML-IAP also reduced the number of BRG1-expressing cells that survived treatment with cisplatin (Figure 3E). Thus, activation of ML-IAP by BRG1 contributes to the previously observed increase in the resistance of BRG1-expressing SK-MEL-5 melanoma cells to this chemotherapeutic agent (Keenen et al., 2010).

As a complementary approach, we transiently expressed an ML-IAP cDNA in cells that lack BRG1 and detected expression of ML-IAP protein. Cleaved PARP was not detected in either control or ML-IAP-expressing cells that had been sham-irradiated (Figure 3F). Upon UV irradiation, cleaved PARP accumulated with similar kinetics as in Figure 1A but was substantially reduced by expression of ML-IAP (Figure 3G). Furthermore, forced ML-IAP expression resulted in an increase in the number of cells that survived exposure to UV radiation (Figure 3H). Thus, activation of ML-IAP by BRG1 contributes to the observed resistance of BRG1-expressing melanoma cells to UV-induced apoptosis.

### Expression of ML-IAP is dependent on coexpression of MITF and BRG1, but not BAF180

**Figure 4:**
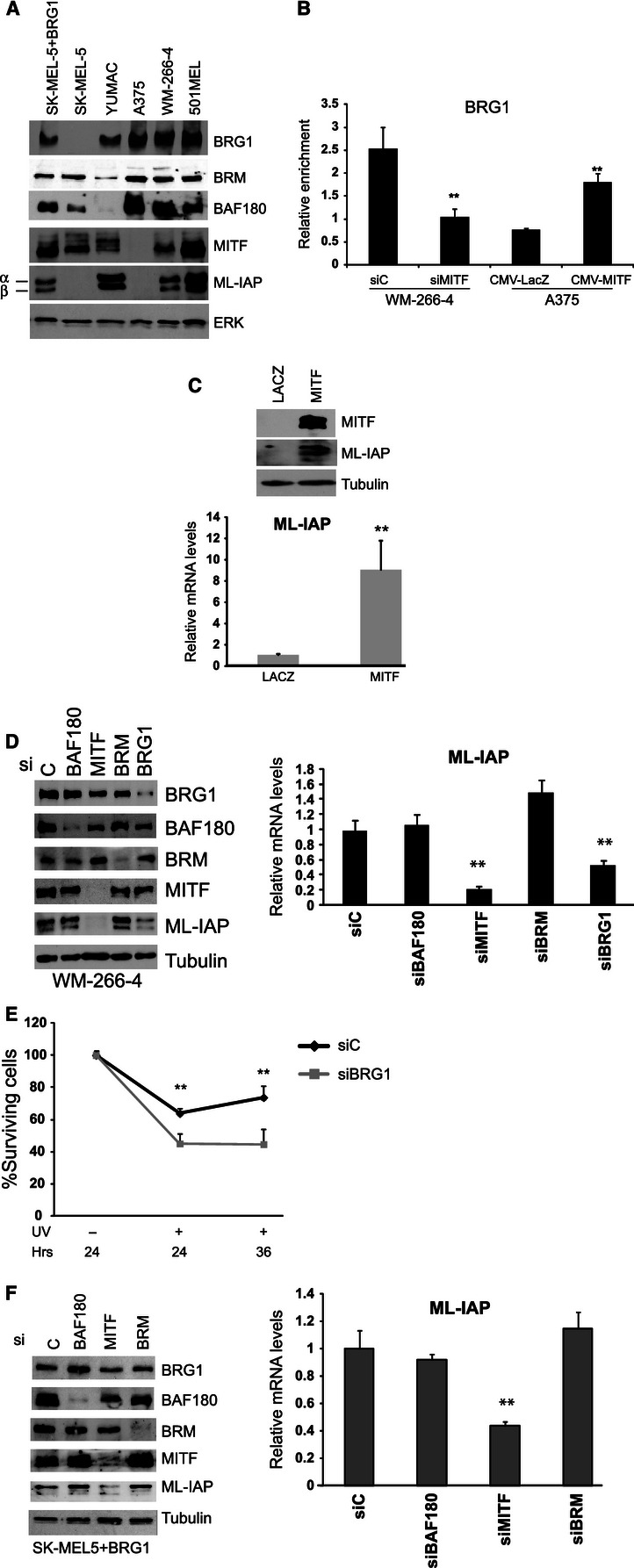
Coexpression of MITF and BRG1 is required for high levels of ML-IAP expression in melanoma cells. (A) Cell extracts from a panel of melanoma cells were subjected to Western blotting with antibodies to BRG1, BRM, BAF180, MITF, and ML-IAP. Total ERK is a loading control. (B) ChIP analysis of the dependency of BRG1 occupancy on the ML-IAP promoter on MITF. WM-266-4 cells were transfected with control siRNA or siMITF, and A375 cells were transfected with CMV-LacZ or CMV-MITF and subjected to ChIP analysis. ChIP enrichment of BRG1 was normalized to IgG and to the control CD25 promoter. BRG1 enrichment on the ML-IAP promoter in WM-266-4 cells was significantly abrogated by siMITF (P < 0.01). BRG1 enrichment on the z promoter was significantly increased in A375 cells by transfection with CMV-MITF (P < 0.01). The data are the average of two independent experiments that were subjected to qPCR twice. Standard error bars are shown. (C) A375 melanoma cells were transfected with CMV-LACZ or CMV-MITF as in Figure 4B. Top: Western blot was performed on A375 cells with antibodies to MITF and ML-IAP. Tubulin is a loading control. The data are representative of three independent experiments. Bottom: mRNA was reverse-transcribed from CMV-LACZ- and CMV-MITF-transfected A375 cells. ML-IAP expression was analyzed by qPCR and normalized to GAPDH. ML-IAP expression was significantly higher in the (z) A375 cells (P < 0.01). The data are the average of two independent experiments subjected to qPCR twice. Standard error bars are shown. (D) WM-266-4 cells were transfected with siControl, si BAF180, siMITF, siBRM, or siBRG1. Left: A Western blot was performed with extracts prepared from control and knockdown cells and probed with the indicated antibodies. Tubulin is a loading control. The data are representative of two independent experiments. Right: mRNA was reverse-transcribed from control and knockdown cells. ML-IAP expression was analyzed by qPCR and normalized to RPL9. MITF and BRG1 depletion significantly inhibited ML-IAP expression (P < 0.01). The data are the average of two or more independent experiments subjected to qPCR twice. Standard error bars are shown. (E) WM-266-4 cells were transfected with a control siRNA or siBRG1, UV-irradiated, and counted at the indicated time points. Percent survival is relative to corresponding sham-irradiated cells taken at 24 h. Percent survival was significantly lower in BRG1 knockdown cells compared with control cells (P < 0.01). The data are the average of two independent experiments performed in triplicate. Standard deviations are shown. (F) SK-MEL-5 cells expressing BRG1 were transfected with siControl, si BAF180, siMITF, or siBRM. Left: A Western blot was performed with extracts prepared from control and knockdown cells and probed with the indicated antibodies. Tubulin is a loading control. Right: mRNA was isolated and reverse-transcribed from control and knockdown cells. ML-IAP expression was analyzed by qPCR and normalized to RPL9. MITF depletion significantly inhibited ML-IAP expression compared with control (P < 0.01). Standard error bars are shown.

ML-IAP has a restricted range of expression, being highly expressed in melanoma cells that express MITF and in some additional cancer cell lines (Dynek et al., 2008; Kasof and Gomes, 2001). We found that in a panel of melanoma cell lines, ML-IAP expression was correlated with coexpression of both MITF and BRG1 (Figure 4A). A375 melanoma cells express high levels of BRG1, but low levels of MITF and undetectable levels of ML-IAP, whereas SK-MEL-5 cells express high levels of MITF, but virtually undetectable levels of BRG1 and undetectable levels of ML-IAP. However, there was no correlation between ML-IAP expression and expression of the BRG1-associated factor, BAF180, nor between ML-IAP expression and expression of the alternative SWI/SNF ATPase, BRM (Figure 4A). BRG1 was significantly enriched on the ML-IAP promoter in WM-266-4 cells, a cell line that expresses high levels of ML-IAP and MITF, compared with A375, cells which express very low levels of MITF (data not shown). Furthermore, the enrichment of BRG1 on the ML-IAP promoter was abrogated by siRNA-mediated knockdown of MITF in WM-266-4 cells and increased by transfection of MITF in A375 cells (Figure 4B). Thus, occupancy of BRG1 on the ML-IAP promoter is dependent on expression of MITF in these melanoma cells.

We further investigated the corequirement for MITF and SWI/SNF components in the activation of ML-IAP in a series of overexpression and knockdown experiments. Transient overexpression of MITF in melanoma cells that are deficient in MITF (A375) but that express high levels of BRG1 activates ML-IAP expression (Figure 4C). Furthermore, in WM-266-4 cells, depletion of BRG1 and MITF by RNA interference substantially reduced ML-IAP protein (Figure 4D, left) and significantly decreased ML-IAP mRNA levels (Figure 4D, right). Depletion of BAF180 and BRM had small effects on the expression of MITF and BRG1 at the protein level (Figure 4D, left) but did not affect ML-IAP expression at the protein (Figure 4D, left) or at the mRNA level (Figure 4D, right). Thus, BRG1 and MITF are required for ML-IAP expression in these cells. We found that depletion of BRG1 in WM-266-4 significantly decreased the number of cells that survived following UV irradiation (Figure 4E). Likewise, several studies indicate that MITF can promote melanocyte and melanoma survival following UV radiation (Hornyak et al., 2009; Liu et al., 2009). Thus, the corequirement for MITF and BRG1 in the regulation of ML-IAP expression is highly correlated with enhanced survival following UV irradiation in multiple melanoma cell lines.

Similar to the effects of MITF depletion in WM-266-4 cells, depletion of MITF in SK-MEL-5 cells expressing BRG1 decreased expression of ML-IAP at the protein level and mRNA level (Figure 4F). ML-IAP expression was not affected by depletion of either BAF180 or BRM (Figure 4F). Thus, in melanoma, BRG1 and MITF activate ML-IAP, independently of BAF180. BRM contributes to ML-IAP regulation when BRG1 is absent (Keenen et al., 2010) but does not fully compensate for BRG1 loss.

**Figure 5:**
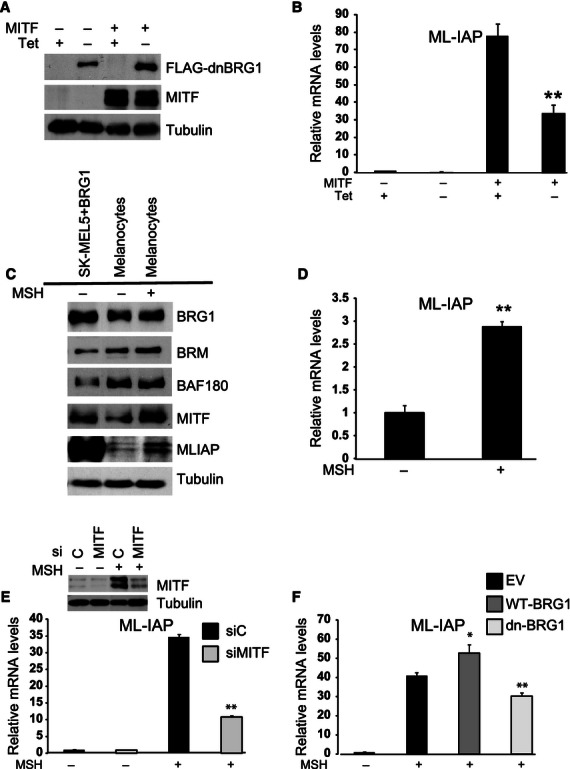
MITF and BRG1 activate ML-IAP expression in a tissue culture model of melanocyte differentiation and in human and mouse melanocytes. (A) Cell lines (B22) that stably express dominant-negative BRG1 (FLAG-tagged) were infected with either control retrovirus or retrovirus expressing MITF in the presence or absence of tetracycline and then cultured in low serum media. Cell extracts were subjected to Western blotting with antibodies to MITF and FLAG. Tubulin is a loading control. (B) cDNA from control B22 cells or cells expressing MITF that were cultured in the presence or absence of tetracycline (Tet) was subjected to qPCR with ML-IAP primers and normalized to RPL7. ML-IAP expression in cells cultured without tetracycline (dominant-negative BRG1 on) was significantly decreased (P < 0.01) compared with ML-IAP expression in cells cultured with tetracycline (dominant-negative BRG1 off). The data are the average of two independent experiments that were subject to qPCR twice. Standard error bars are shown. (C) Cell extracts from SK-MEL-5 cells expressing BRG1 and neonatal epidermal melanocytes (untreated or treated with NDP-MSH for 24 h) were subjected to Western blotting with antibodies to BRG1, BRM, BAF180, MITF, and ML-IAP. Tubulin is a loading control. (D) cDNA from control human epidermal melanocytes or melanocytes treated with NDP-MSH (24 h) was subjected to qPCR with ML-IAP primers and normalized to RPL9. (E) Immortalized mouse melanoblast (Melb-a) cells were transfected with siControl or siMITF and cultured in the presence or absence of NDP-MSH for 24 h. Cell extracts were subjected to Western blotting with an antibody to MITF. Tubulin is shown as a loading control (Top). cDNA was subjected to qPCR with primers to ML-IAP and normalized to RPL7. The data are representative of two experiments performed in triplicate. (F) Melb-a cells as in E were transfected with an empty vector (EV), wild-type BRG1(WT-BRG1), or dominant-negative BRG1 (dn-BRG1). cDNA was subjected to qPCR with primers to ML-IAP and normalized to RPL7. The data are representative of two experiments performed in triplicate.

Although the requirement for MITF has been investigated in melanoma cells, it is not known whether MITF regulates ML-IAP expression in non-tumorigenic cells. By utilizing a tissue culture model of melanocyte differentiation (de la Serna et al., 2006a), we found that MITF activated ML-IAP expression and that activation of ML-IAP was abrogated by a dominant-negative version of BRG1 (Figure 5A, B). Thus, ML-IAP is activated in a lineage-specific manner in non-tumorigenic cells by a mechanism that is dependent on BRG1.

To determine whether ML-IAP expression in normal melanocytes is dependent on UV-exposure, we investigated the expression of ML-IAP in melanocytes that were exposed to a stable analog of alpha-melanocyte-stimulating hormone (MSH). On UV-irradiated skin, keratinocytes synthesize MSH, a ligand for the melanocortin-1 receptor (MC1R) located on the melanocyte surface (Rouzaud et al., 2005). Binding of MSH to MC1R activates the cyclic AMP pathway and promotes MITF expression in melanocytes. ML-IAP was induced by treatment of primary human melanocytes with a stable analog of MSH (Figure 5C, D). These data are consistent with a previous report that detected an increase in ML-IAP expression in forskolin-treated melanocytes (Dynek et al., 2008).

Although ML-IAP was induced by MSH in melanocytes, the level of ML-IAP protein was substantially lower in induced human melanocytes than in SK-MEL-5 cells that express BRG1 (Figure 5C). Thus, additional factors are likely involved in the regulation of ML-IAP in melanoma cells because both MITF and BRG1 are expressed at similar levels in induced melanocytes as in melanoma cells that express higher levels of ML-IAP. Interestingly, expression of BAF180 was higher in melanocytes than in SK-MEL-5 cells expressing BRG1 (Figure 5C). Furthermore, BAF180 was depleted in parental SK-MEL-5 and YUMAC melanoma cells compared with other melanoma cells (Figure 4A). Thus, the relative association of BRG1 with BAF- versus PBAF-specific subunits in melanocytes may be different from that in a subset of melanoma cells, potentially contributing to the differential wiring of melanocytes and melanoma cells.

To determine the requirement of MITF and BRG1 in the regulation of ML-IAP in normal melanocytic cells, we introduced siRNA that targets MITF in mouse melanoblasts (Figure 5E, top). MSH promoted an increase in ML-IAP expression, which was abrogated by down-regulation of MITF (Figure 5E, bottom). Furthermore, ectopic expression of wild-type BRG1 enhanced ML-IAP expression in MSH-activated melanoblasts, whereas a dominant-negative version of BRG1 significantly decreased ML-IAP expression (Figure 5F). Thus, ML-IAP expression is likely to be activated by UV radiation when melanocytes are in their natural microenvironment by a mechanism that is dependent on MITF and BRG1.

### BRG1 regulates ML-IAP expression by cooperating with MITF to alter chromatin structure on the ML-IAP promoter

**Figure 6:**
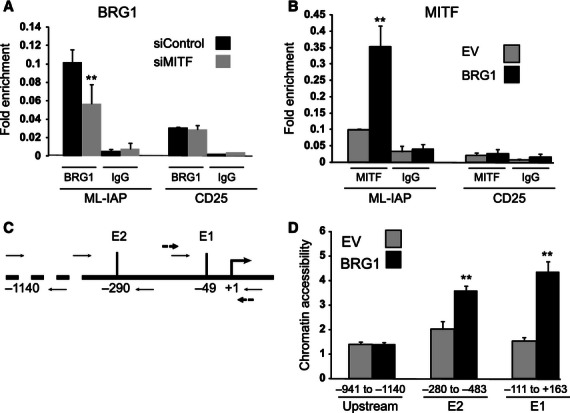
BRG1 regulates ML-IAP expression by cooperating with MITF to remodel chromatin structure. (A) SK-MEL-5 cells expressing BRG1 were transfected with scrambled siRNA or siRNA that targets MITF. Fold enrichment of BRG1 or a control IgG was normalized to input at the ML-IAP promoter and control CD25. Down-regulation of MITF resulted in a significant reduction (P < 0.05) in the enrichment of BRG1 at the ML-IAP promoter. The data are representative of three independent experiments and the average of one experiment that was subjected to qPCR twice (in triplicate). Standard error bars are shown. (B) ChIPs were performed with control SK-MEL-5 cells (EV) and SK-MEL-5 cells expressing BRG1 using an antibody specific for MITF or control IgG. Enrichment of MITF or IgG at the ML-IAP promoter and control CD25 promoter is expressed relative to input. Fold enrichment of MITF or a control IgG was normalized to input at the ML-IAP promoter and control CD25. There was a significant increase in MITF binding to the ML-IAP promoter (P < 0.01) in BRG1-expressing SK-MEL-5 cells compared with EV-expressing cells. Similar results for MITF were obtained with primers that encompass Ebox2 (data not shown). The data are the average of three independent experiments. Standard error bars are shown. (C) Schematic of the ML-IAP promoter illustrates the position of the two E boxes and the position of the primers (solid arrows) for analysis of chromatin accessibility experiments and (dashed arrows) for analysis of ChIP experiments. (D) Nuclei were isolated from control SK-MEL-5 cells (EV) or SK-MEL-5 cells expressing BRG1 and digested with MNase I. Accessibility was calculated (*CT*_undigested_–*CT*_digested_) by normalizing to undigested samples. Accessibility at Ebox1 and Ebox2 was significantly increased in BRG1-expressing cells (P < 0.01). Accessibility at an upstream region (−941 to −1140) was not significantly affected by BRG1. The data are the average of two independent experiments that were subjected to qPCR twice. Standard error bars are shown.

SWI/SNF enzymes are recruited to promoters by interactions with gene-specific transcriptional activators (de la Serna et al., 2006b). However, transcriptional activators have limited access to their binding sites when embedded in repressive chromatin structure. We found that MITF is required to recruit BRG1 to the ML-IAP promoter (Figures 4B and 6A) as well as other MITF-regulated genes (Keenen et al., 2010). Interestingly, we found that MITF binding to the ML-IAP promoter also requires BRG1, suggesting that recruitment of MITF and BRG1 to the ML-IAP promoter is interdependent (Figure 6B).

To understand the mechanism by which MITF promotes the recruitment of BRG1 concomitantly with a requirement for BRG1 in promoting MITF recruitment, we performed chromatin accessibility experiments to probe the changes in chromatin structure elicited by BRG1 on the ML-IAP promoter. The ML-IAP promoter has two E boxes, both of which bind MITF and are activated by MITF (Dynek et al., 2008). We assayed BRG1-induced changes in chromatin structure on the ML-IAP promoter by digesting nuclei from control and BRG1-expressing SK-MEL-5 cells with micrococcal nuclease (MNase I). We then utilized a CHART-PCR assay (Rao et al., 2001) to detect changes in accessibility to MNase I at regions encompassing each of the E boxes and at an upstream region of the ML-IAP promoter (Figure 6C).

The regions surrounding both E boxes were more accessible to MNase I in BRG1-expressing cells, while a 5′ upstream region was unaffected by BRG1 (Figure 6D). The accessibility of the region surrounding Ebox1 was increased by BRG1 to a greater extent than that of the region encompassing Ebox2. However, we were not able to resolve differential effects of BRG1 on MITF binding to the two E boxes (data not shown). Thus, our data indicate that MITF has limited accessibility to its recognition sites in the ML-IAP promoter and weakly associates with the promoter at one or both E boxes. Furthermore, the data demonstrate that MITF-dependent recruitment of BRG1 augments MITF interactions with the ML-IAP promoter by increasing the accessibility of its binding sites. Thus, cooperative interactions between MITF and BRG1 promote recruitment of both factors to the ML-IAP promoter.

### BRG1 promotes histone chromatin modifications on the ML-IAP promoter and alters chromatin-modifying enzyme recruitment

**Figure 7:**
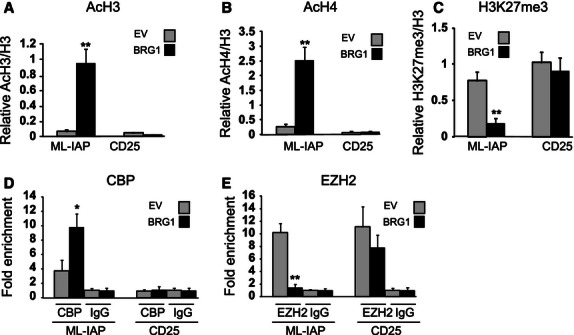
BRG1 promotes histone modifications on the ML-IAP promoter characteristic of active transcription. ChIPs were performed on control SK-MEL-5 cells (EV) and (BRG1) SK-MEL-5 cells. Enrichment on the ML-IAP promoter and on the silent CD25 promoter (control) was assayed by qPCR. ChIP enrichment is expressed relative to histone H3. Use of control IgG in ChIPs resulted in <1% relative enrichment of enrichment with antibodies to the indicated histone modifications (data not shown). (A) ChIP analysis of H3 acetylation (AcH3) relative to histone H3. (B) ChIP analysis of H4 acetylation (AcH4) relative to histone H3. (C) ChIP analysis of H3 lysine 27 tri-methylation (H3K27me3) ChIP enrichment relative to histone H3. (D) ChIP analysis of CBP and control IgG. E. ChIP analysis of EZH2 and control IgG. Statistically significant differences in histone modifications and association of histone-modifying enzymes at the ML-IAP and CD25 promoters in control (EV) and BRG1-expressing cells are indicated. Significant differences are indicated with ** for P < 0.01 and * for P < 0.05. The data are the average of two or more independent experiments subjected to qPCR twice. Standard error bars are shown.

We previously detected a dramatic increase in the levels of the active H3K4me3 mark on the ML-IAP promoter as a result of BRG1 expression in SK-MEL-5 cells (Keenen et al., 2010). To determine the mechanisms by which BRG1 activates transcription of ML-IAP, we assayed whether other chromatin modifications associated with active transcription are modulated as a result of BRG1 expression. We found that BRG1 promoted a significant increase in AcH3 levels (Figure 7A) and in AcH4 levels (Figure 7B) on the ML-IAP promoter. Although the levels of these histone modifications on the ML-IAP promoter were low in cells that lack BRG1, they were significantly higher than on the silent CD25 promoter, suggesting a partially open chromatin configuration when BRG1 is absent.

Transcriptional activation by SWI/SNF enzymes can also involve suppression of inhibitory chromatin covalent modifications such as histone H3 tri-methylation at lysine 27 (H3K27me3). Interestingly, H3K27me3 levels on the ML-IAP promoter were significantly lower in BRG1-expressing cells than in control cells, suggesting that BRG1 disrupts this repressive mark (Figure 7C).

We hypothesized that weak MITF binding to one or both E boxes is sufficient to allow recruitment of a chromatin-remodeling enzyme that maintains a low level of histone acetylation on the ML-IAP promoter, facilitating the recruitment of BRG1. Recruitment of BRG1 is then required to increase the accessibility of MITF-binding sites and MITF association with the ML-IAP promoter and to promote further chromatin modifications required for active transcription. To test this hypothesis, we performed ChIPs to detect chromatin-modifying enzymes likely to elicit the observed histone covalent modifications on the ML-IAP promoter. BRG1 significantly enhanced the association of CBP with the ML-IAP promoter (Figure 7D), indicating that in addition to enhanced MITF binding, recruitment of CBP, an MITF coactivator (Sato et al., 1997), is also enhanced by BRG1.

SWI/SNF and polycomb complexes have antagonistic functions during embryonic development and in oncogenic transformation (Wilson et al., 2010). Because SWI/SNF complexes can mediate the eviction of polycomb-silencing complexes that catalyze H3K27 tri-methylation (Kia et al., 2008), we explored the possibility that reduction in H3K27me3 on the ML-IAP promoter was due to eviction of the polycomb component, EZH2. Interestingly, in control cells (EV), ChIP enrichment of EZH2 on the ML-IAP promoter was not significantly different from EZH2 enrichment on the CD25 promoter (Figure 7E). BRG1 disrupted the association of EZH2 with the ML-IAP promoter but did not have a significant effect on the association of EZH2 with the CD25 promoter.

**Figure 8:**
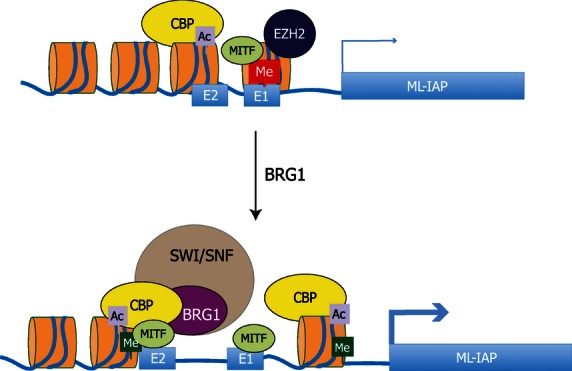
Hypothetical model for BRG1-mediated activation of ML-IAP transcription.

In combination, these data suggest that in the absence of BRG1, the ML-IAP promoter is in a partially open chromatin configuration characterized by low levels of histone acetylation and high levels of H3K27 tri-methylation. BRG1 renders MITF sites fully accessible, thereby promoting increased recruitment of CBP and high levels of histone acetylation. Recruitment of BRG1 to the ML-IAP promoter also disrupts the association of EZH2 and generates chromatin signature consistent with transcriptional activation (Figure 8). Thus, we demonstrate that antagonism between SWI/SNF complexes and a component of the polycomb complex in cancer cells extends beyond the regulation of tumor suppressor genes (Kia et al., 2008) and includes that of a prosurvival gene.

## Discussion

Microphthalmia-associated transcription factor (MITF) is essential for melanocyte development and for the survival of both melanocytes and melanoma cells. MITF activity can also influence melanocyte survival in response to UV radiation and melanoma resistance to chemotherapeutics (Hornyak et al., 2009). MITF is considered a lineage addiction oncogene based on the ongoing requirement for MITF in melanoma tumorigenicity and contribution to melanoma chemoresistance (Garraway et al., 2005). The ability of MITF to act as a prosurvival factor in melanoma is thought to partially rely on the transcriptional regulation of target genes that include two inhibitors of apoptosis: BCL2 and ML-IAP.

ML-IAP is a member of the conserved inhibitor of apoptosis (IAP) family that is highly expressed in melanoma and correlates with aggressive behavior, poor response to chemotherapeutic agents, and decreased survival (Lazar et al., 2012). The inhibitor of apoptosis family of proteins (IAPs) includes proteins that contain one or more repeats of a 70 amino acid domain, termed ‘the baculoviral IAP repeat’ (BIR), and may also contain a RING motif. The IAPs have diverse functions that include eukaryotic development, cell cycle regulation, and inhibition of programmed cell death. Inhibition of cell death occurs in response to diverse proapoptotic stimuli by either direct or indirect inhibition of caspase activity (O'Riordan et al., 2008).

Interestingly, ML-IAP is markedly up-regulated in melanoma cells compared with normal melanocytes and promotes tumorigenicity; however, disruption of ML-IAP does not have an effect on mouse development or survival of the melanocyte lineage (Varfolomeev et al., 2012). The selective requirement for ML-IAP in cancer cells has identified it as an attractive target for cancer treatment (Flygare et al., 2012).

Our current data indicate that in normal melanocytes, ML-IAP expression is up-regulated by MSH, a factor that is activated in response to ultraviolet radiation and that MITF and the BRG1 component of the SWI/SNF complex are required to activate ML-IAP expression in response to MSH. Thus, although additional mechanisms likely promote the overexpression of ML-IAP in melanoma cells, its regulation is lineage dependent, originating in normal melanocytes.

In this study, we determined that BRG1 protects melanoma cells from apoptosis following UV irradiation by cooperating with MITF to regulate ML-IAP transcription. Transcriptional regulation of ML-IAP involves SWI/SNF-mediated disruption of nucleosome structure and modulation of the association of other chromatin-modifying enzymes. Thus, a specific subunit of the SWI/SNF complex, BRG1, determines which of its potential target genes, a lineage-specific oncogene such as MITF activates. Interestingly, high-throughput chromatin analysis combined with genome-wide ChIPs indicated that MITF-occupied sites are surrounded by positioned nucleosomes, suggesting that MITF binds preferentially to nucleosome-free regions and/or promotes nucleosome-free regions (Ozsolak et al., 2007). However, the mechanisms that establish and maintain the precise chromatin conformation required for MITF to activate specific target genes in melanoma cells were not previously known. Our data indicate that MITF and BRG1 containing SWI/SNF complexes coordinately promote transcriptionally permissive chromatin structure on one MITF target, the ML-IAP promoter in melanoma cells.

As an activator of ML-IAP expression in melanoma cells, BRG1 diverges from its well-known role in tumor suppression and promotes a critical survival pathway by cooperating with MITF. Thus, in some melanoma cells, there may be selective pressure for retention of BRG1, as suggested by the overexpression of BRG1 in patient-derived primary melanoma and metastatic melanoma samples (Lin et al., 2010; Saladi et al., 2010). Our data indicate that the activation of ML-IAP does not require the PBAF-specific component, BAF180. BAF180 has been implicated as a tumor suppressor that is mutated in breast and renal cell carcinomas (Varela et al., 2011; Xia et al., 2008) The lack of coordination between BRG1 and BAF180 expression in a subset of melanoma cell lines may cause down-regulation of the tumor-suppressive function of BRG1 while retaining its function in promoting lineage-specific survival. Interestingly, two genome-wide sequencing studies reported that mutations in ARID2, another PBAF component, occur in melanoma at a significant frequency and may be driver mutations (Hodis et al., 2012; Krauthammer et al., 2012). Given the demonstrated role of BRG1 in the repair of UV-induced damage and in the repair of cisplatin cross-links (Kothandapani et al., 2012; Zhao et al., 2009), it is highly likely that there are multiple mechanisms by which SWI/SNF complexes promote enhanced survival of melanocytes and melanoma cells following DNA damage. Elucidation of the requirements for PBAF complexes in regulating SWI/SNF specificity in transcriptional and DNA repair functions will give insight into how SWI/SNF function is deregulated in melanoma.

## Methods

### Cell culture

SK-MEL-5, WM-266-4, and A375 melanoma cells were from the ATCC. 501Mel and YUMAC melanoma cell lines were from Yale Cell Culture Core Facility (New Haven, CT, USA). Melb-a cells were from Dr. Dorothy Bennett (The Welcome Trust). Melanoma cells were cultured as described (Keenen et al., 2010). B22 experiments were performed as described (de la Serna et al., 2006a).Epidermal melanocytes were isolated as described (Liu et al., 2001), cultured in Media 254 (Invitrogen, Carlsbad, CA, USA) and induced with 1 nM NDP-MSH for 24 h (Sigma-Aldrich, St. Louis, MO, USA). Melb-a cells were cultured as described and induced with 2 nM NDP-MSH for 24 h (Sviderskaya et al., 2001).

### UV-irradiation

Melanoma cells were irradiated with a lamp that emits 75% in the UVB range and 25% in the UVA range (National Biologics, Twinsburg, OH, USA) at a dose of 50 mJ/cm^2^. UVC was blocked with a Kodacel sheet (Eastman Kodak, Rochester, NY, USA).

### Transfections

Cells were transfected with Lipofectamine LTX (Invitrogen). SK-MEL-5 cells were transfected with pcDNA2-ML-IAP (Vucic et al., 2000). A375 cells were transfected with CMV-LacZ or CMV-MITF using Lipofectamine LTX (Invitrogen). Melb-a cells were transfected with an empty pBABE, pBABE-WT-BRG1, or pBABE-dnBRG1.

### RNA isolation and quantitative real-time PCR

cDNA was prepared as described (Keenen et al., 2010). Quantitative (q) PCR was performed and analyzed as described (Keenen et al., 2010). Primers for human ML-IAP and GAPDH were from SABiosciences (Qiagen, Valencia, CA, USA). Primers for human RPL9 were 5′AAACAAGCGGATTCTCATGG-3′ and 5′-TTGGTCTCTTCCTCCTTGGAT-3′. Primers for mouse RPL7 were 5′-GGAGGAAGCTCATCTATGAGAAGG-3′ and 5′-AAGATCTGTGGAAGAGGAAGGAGC-3′. Primers for mouse ML-IAP were 5′-GGCCAGCTTCGGCCTCTGTC-3′ and 5′-GGGTCATCCCCACGCTCCCA-3′. Primers for FLAG-BRG1 were 5′-TTTGTCATCGTCGTCCTTGTAGTC-3′ and 5′-GTACAAGGACAGCAGCAGTGGA-3′. A no RT control was included for each primer set.

### Cell extracts and immunoblot analysis

Western blots were performed as described (de la Serna et al., 2000). The tubulin antibody was from Sigma. The BRG1 antiserum was previously described (de la Serna et al., 2006a). The BRM and ML-IAP (livin) antibodies were from Abcam (Cambridge, MA, USA). The BAF180 antibody was from Bethyl Labs (Montgomery, TX, USA). The p21CIP1/WAF1 and p53 antibodies were from Santa Cruz Inc. (Santa Cruz, CA, USA). The caspase 3 and cleaved PARP antibodies were from Cell Signaling Technology (Boston, MA, USA). The MITF (C5) antibody was from Dr. David Fisher (Massachusetts General Hospital, Boston, MA, USA).

### TUNEL assay

The TUNEL assay was performed with the In situ Cell Death Detection kit, Fluorescein (Roche Applied Science, Indianapolis, IN, USA) according to the manufacturer's directions and visualized with a Nikon Eclipse TE2000-U fluorescence microscope (Nikon, Melville, NY, USA).

### Annexin V assay

Cells were stained with the Guava Nexin Annexin V Reagent kit (Millipore, Billerica, MA, USA) and assayed on a Guava Personal Cell Analysis System and analyzed with the Guava Cytosoft software (Millipore).

### Cell counts

Cells were trypsinized and resuspended in media at the indicated times. Cell counts were taken using the Sceptor 2.0 handheld automated cell counter (Millipore).

### siRNA knockdowns

siRNA sequences were as previously described (Burrows et al., 2010; Carreira et al., 2006; Crnkovic-Mertens et al., 2003, 2006; Flowers et al., 2009; Xu et al., 2007; Ye et al., 2011): siControl, 5′-(UUCUCCGAACGUGUCACGU)-3′; siMITF, 5′-(AGCAGUACCUUUCUACCAC)-3′; siBRG1, 5′-(AACAUGCACCAGAUGCACAAG)-3′; siBRM, 5′-(GUCAUAAGCCUGAGGCAAA)-3′; siBAF180, 5′-(GCCGUGUGCCAUGAACUCUAUA)-3′. The siRNA targeting both isoforms of ML-IAP was 5′-(GGAAGAGACUUUGUCCACA)-3′, and the siRNA targeting ML-IAP-α was 5′-(GGGCGUGGUGGGUUCUUGA)-3′. siRNAs were synthesized by Dharmacon (Lafayette, CO, USA) and transfected according to the manufacturer's instructions. Cells were UV-irradiated 72 h after siRNA transfection and assayed by Western blotting or subjected to the annexin V and TUNEL assays, 36 h after irradiation.

### Chromatin immunoprecipitations

Chromatin immunoprecipitations were performed as described in Keenen et al. (2010). Antibodies to histone H3 (cat#39163), AcH3 (cat#39139), and tetra-AcH4 (cat#39179) were from Active Motif (Carlsbad, CA, USA). Control IgG and antibodies to H3K27me3 (cat#ABE44) and EZH2 (cat#17662) were from Millipore. The antibody to MITF (Ab12035) used in ChIPs was from Abcam: forward: 5′-CCTTCCCGTCTTGTTCAGAG-3′ and reverse: 5′-GACAGCAGGGATAGGCACAG-3′ (ML-IAP promoter); and forward: 5′-TTCTTGGTAAGAAGCCGGGAAC-3′ and reverse: 5′-TCCTCTTCAACGGCGAAATTGC-3′ (CD25 promoter).

### Chromatin accessibility

Nuclei were digested with 3 U/ml MNase I (Worthington Biochemical Corp., Lakewood, NJ, USA) for 15 min. Purified genomic DNA was subjected to qPCR and analyzed as described in Cruickshank et al. (2008) and Rao et al. (2001). Primers for qPCR were the following: forward: 5′-ACAGAGCATGTGACCCCAGA-3′and reverse: 5′-ACCAGGTTTGCAGCAGAAAT-3′ (E box 1, −111 to +163); forward: 5′-TCTGATCTTCCTGGCCTGAG-3′ and reverse: 5′-AGGACATGTGAGCTGTGCTG-3′ (E box 2, −280 to −483); and forward: 5-GCAGGTGTGAAAGTGTGGTG-3′ and reverse: 5′-GTGCAGGCTCACAGAGTTTG-3′ (Upstream, −941 to −1140).

### Statistical analysis

Statistical significance was calculated by the student's *t*-test (two groups) and by anova (three or more groups).
